# Variation in Alpha-Case Thickness of Ti-xAl Castings

**DOI:** 10.3390/ma19010029

**Published:** 2025-12-21

**Authors:** Byungil Kang, Taekyu Ha, Seul Lee, Youngkyu Ju, Youngjig Kim

**Affiliations:** 1 Department of Advanced Materials Science and Engineering, Sungkyunkwan University, 2066 Seobu-ro, Jangangu, Suwon 16419, Gyeonggi-do, Republic of Korea; subs39th@skku.edu (B.K.); taekyu@skku.edu (T.H.); 2 Materials Engineering Team, Hanwha Aerospace, 6, Pangyo-ro, 319beon-gil, Bundang-gu, Seongnam-si 13488, Gyeonggi-do, Republic of Korea; seul.lee@hanwha.com (S.L.); yk.ju@hanwha.com (Y.J.)

**Keywords:** titanium alloy, aluminum content, alpha-case formation, investment casting, oxygen diffusion

## Abstract

Alpha-case formation, originating from interfacial reactions between molten titanium and oxide molds, remains a critical issue limiting the surface integrity and mechanical performance of titanium castings. In this study, the effect of aluminum content (0–52 at%) on alpha-case formation was systematically investigated using plasma arc melting and drop casting with alumina-based molds. The reaction kinetics between titanium melts and alumina molds were evaluated through cooling rate measurements and thermodynamic modeling. Microstructural and compositional analyses using optical microscopy, hardness testing, and electron probe microanalysis revealed that increasing aluminum content effectively suppressed alpha-case development. No distinct reaction layer was observed when the aluminum concentration exceeded 30 at%. The alpha-case consisted primarily of Ti_3_Al, TiO_2_, and Ti_5_Si_3_ phases, indicating that the molten titanium reacted with both alumina and silica constituents of the mold. Oxygen was identified as the dominant element controlling the reaction depth, consistent with its diffusion behavior across titanium phases. Calculated alpha-case thicknesses showed excellent agreement with experimental measurements, confirming that the reduction in alpha-case depth with increasing aluminum content results from decreased oxygen diffusivity, shorter reaction time, and lower interfacial temperature. These findings establish aluminum addition as a key strategy for minimizing interfacial reactions during titanium investment casting, thereby improving dimensional accuracy and surface quality in high-temperature components.

## 1. Introduction

Titanium alloys have been widely used in aerospace, energy, and biomedical applications owing to their high specific strength, corrosion resistance, and superior high-temperature performance. Among various alloy systems, alpha-type and near-alpha titanium alloys are particularly attractive due to their excellent creep resistance, oxidation resistance, and weldability compared to beta-type alloys. Aluminum is known as the most effective alpha-phase stabilizer, improving oxidation and creep resistance at elevated temperatures. Consequently, aluminum-containing titanium alloys such as Ti-6Al-2Sn-4Zr-2Mo have been extensively employed for compressor blades and disks operating at temperatures up to 565 °C [1,2,3]. These alloys have therefore been increasingly utilized in advanced turbine components for high-temperature service, where the demand for both weight reduction and high strength is critical. Such trends align with the broader development of γ-TiAl-based intermetallic as next-generation lightweight materials offering comparable high-temperature capability to nickel-based superalloys [4].

However, despite these advantages, the processing of titanium alloys remains challenging because of their high reactivity in the molten state and their limited workability. In particular, during investment casting, molten titanium readily reacts with oxide molds, forming a brittle oxygen-enriched surface layer known as the alpha-case [5,6,7]. This reaction layer deteriorates mechanical properties by acting as a crack initiation site and by reducing fatigue strength and ductility. Therefore, post-casting treatments such as machining, chemical milling, or pickling are often required to remove the affected region [7,8]. The ability to minimize alpha-case formation is thus critical to achieving net-shape titanium components with high dimensional accuracy and reliability.

Alpha-case development is known to depend on several factors, including casting temperature, alloy composition, cooling rate, and mold materials. Among these, aluminum plays a particularly important role, as it not only stabilizes the alpha phase but also influences oxygen solubility and diffusivity in the titanium matrix [9,10,11,12,13]. Recent efforts to develop non-reactive mold systems for Ti-48Al-2Cr-2Nb alloys have demonstrated the critical role of aluminum in controlling interfacial reactivity and oxygen diffusion [14]. Nevertheless, the quantitative relationship between aluminum content and the suppression of alpha-case formation across different titanium alloys remains insufficiently understood.

Accordingly, the present study aims to elucidate the effect of aluminum content on the formation and growth behavior of alpha-case layers in titanium alloys. Through combined thermodynamic and diffusion analyses, the influence of aluminum on interfacial reactivity, oxygen transport, and phase evolution during titanium casting was systematically investigated. The results provide fundamental insights into controlling surface reactions and optimizing casting processes for high-temperature titanium components.

## 2. Experimental Procedure

### 2.1. Mold Preparation

Alumina (Al_2_O_3_, 99% purity, 325 mesh; Union Co., Ltd., Seoul, Republic of Korea) and colloidal silica (30 wt% SiO_2_, 15 nm particles; Serin Hitech, Seoul, Republic of Korea) were used as the primary coating materials for the investment molds. Colloidal silica was added to the slurry to achieve an efflux time of 40 s, measured using a Zahn cup #4 (ASTM D4212 standard [15]), ensuring consistent slurry viscosity. The primary coating was applied twice to achieve a uniform surface layer.

For the backup layers, chamotte (Al_2_O_3_·SiO_2_, 98% purity, 325 mesh; Dowon Mulsan Co., Ltd., Pyeongtaek, Republic of Korea) and colloidal silica were used, with Al_2_O_3_ stucco (99% purity, 18–32 mesh; Union Co., Ltd., Seoul, Republic of Korea) applied as the refractory material. The backup coating was applied three times at 4 h intervals to enhance mold strength. The completed molds were dewaxed in an autoclave at 150 MPa and subsequently fired in air at 1223 K for 2 h to remove residual binders and stabilize the shell structure.

### 2.2. Alloy Fabrication and Casting

**Figure 1:**
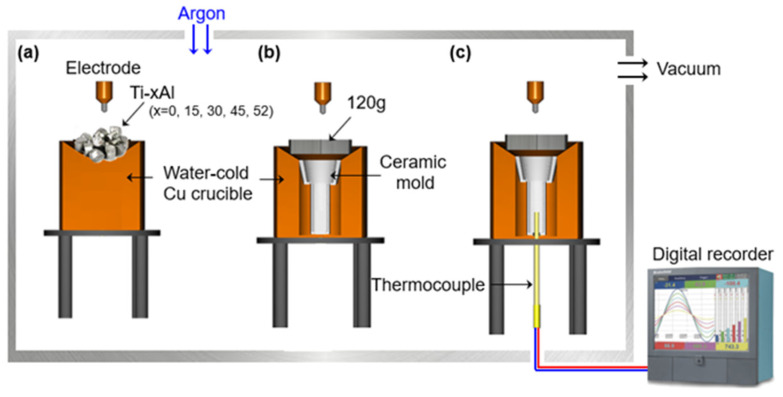
Schematic illustration of the plasma arc melting (PAM) and dropcasting process: (**a**) alloying of Ti-xAl alloys, (**b**) drop-casting procedure, and (**c**) setup for measuring the cooling rate. The thermocouple recorded the temperature only after the molten alloy was poured into the mold.

Titanium alloys with different aluminum contents (0, 15, 30, 45, and 52 at%) were prepared using a plasma arc melting (PAM) furnace. Each alloy was remelted four times under an argon atmosphere to ensure chemical homogeneity. Approximately 120 g of alloy was then placed on a water-cooled copper hearth, and the preheated mold was positioned above the melt for drop casting. The overall alloying and casting setup is illustrated schematically in Figure 1. Although the thermocouple was inserted into the interior of the ceramic mold, the temperature was recorded only after the molten alloy was poured into the mold positioned inside the water-cooled copper crucible. Thus, the thermocouple captured the temperature evolution during and after the onset of solidification rather than the initial superheat of the melt.

### 2.3. Characterization

**Figure 2:**
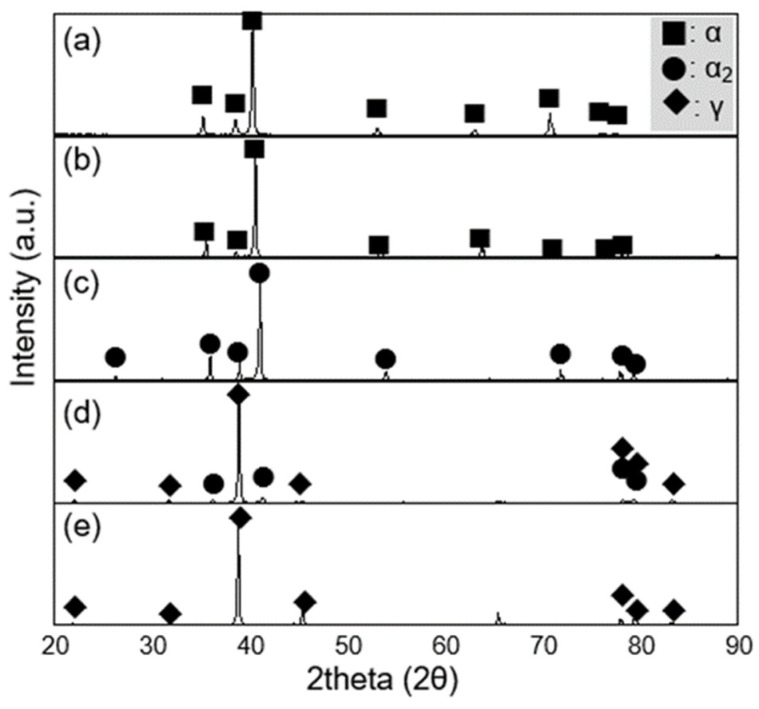
X-ray diffraction (XRD) patterns of Ti-xAl alloys with various aluminum contents: (**a**) cp-Ti, (**b**) Ti-15Al, (**c**) Ti-30Al, (**d**) Ti-45Al, and (**e**) Ti-52Al.

The phase constituents of the cast alloys were identified by X-ray diffraction (XRD, M18XHF-SRA, Mac Science Co., Ltd., Yokohama, Japan). The phase fractions presented in Figure 2 were estimated using a semi-quantitative method based on the integrated peak areas of the major diffraction peaks for α, α_2_, and γ. After background subtraction, the relative intensity ratio method was applied to approximate the weight fractions. The alpha-case thickness was determined using optical microscopy (OM, PME-3, Olympus Corporation, Tokyo, Japan) and micro-Vickers hardness testing (MVK-H2, Mitutoyo Corporation, Kawasaki, Japan) to evaluate both microstructural and mechanical variations from the surface. Hardness profiles were measured from 1000 μm below the surface toward the exterior with a 100 g load applied at 50 μm intervals.

The chemical composition and phase distribution within the alpha-case were examined using electron probe microanalysis (EPMA, JXA-8500F, JEOL Ltd., Tokyo, Japan) and high-resolution transmission electron microscopy (HRTEM, JEM-3010, JEOL Ltd., Tokyo, Japan). The cooling rate of the castings was monitored by a B-type thermocouple connected to a digital recorder (PR2006, CHINO, Tokyo, Japan) to estimate the effective reaction time between the molten alloy and the oxide mold interface.

## 3. Results and Discussion

### 3.1. Phase Identification of Titanium Alloys

The phase constituents of Ti-xAl alloys fabricated via plasma arc melting (PAM) are shown in Figure 2. In commercially pure titanium (cp-Ti) and Ti–15Al, the structure consisted predominantly of α-Ti (approximately 99%) with less than 1% β phase. In Ti-30Al, the dominant phase was α_2_ (94%) with a minor α fraction. As aluminum content increased further, the γ phase fraction increased significantly–reaching about 76% in Ti-45Al and nearly 100% in Ti-52Al.

These results clearly demonstrate the phase evolution sequence of α → α_2_ → γ with increasing aluminum concentration, which is consistent with the Ti-Al binary phase diagram [16]. The stabilization of the γ phase at higher aluminum contents suggests that aluminum strongly promotes the ordered L1_0_ structure, leading to improved high-temperature stability of the alloy system.

### 3.2. Variation in Alpha-Case Thickness

**Figure 3:**
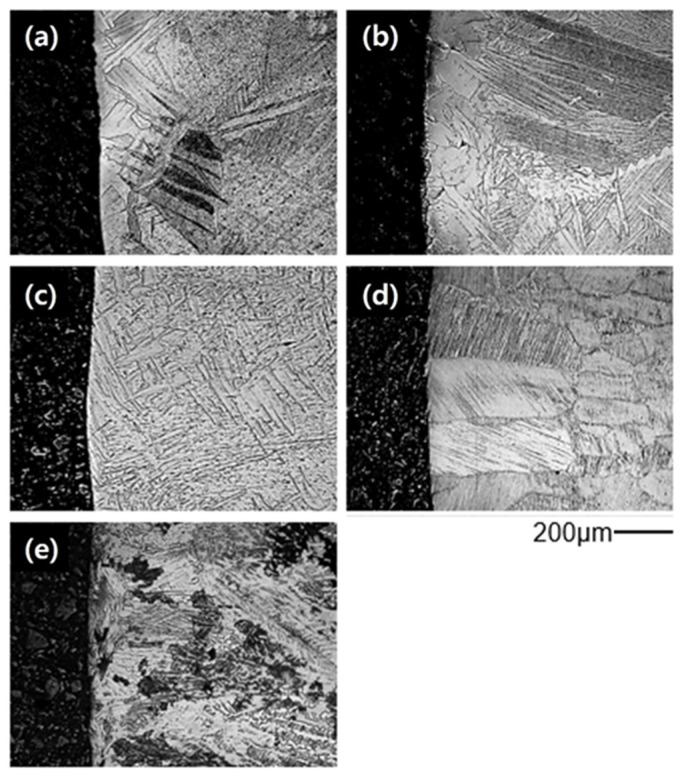
Surface microstructures of Ti-xAl alloys showing the suppression of alpha-case formation with increasing aluminum content: (**a**) cp-Ti, (**b**) Ti-15Al, (**c**) Ti-30Al, (**d**) Ti-45Al, and (**e**) Ti-52Al.

Figure 3 shows the surface microstructures of Ti-xAl castings. Widmanstätten structures were observed in cp-Ti and Ti-15Al, indicating the formation of oxygen-enriched alpha-case layers near the surface. In contrast, alloys containing more than 30 at% aluminum exhibited uniform microstructures without visible surface reaction layers, implying effective suppression of alpha-case formation.

**Figure 4:**
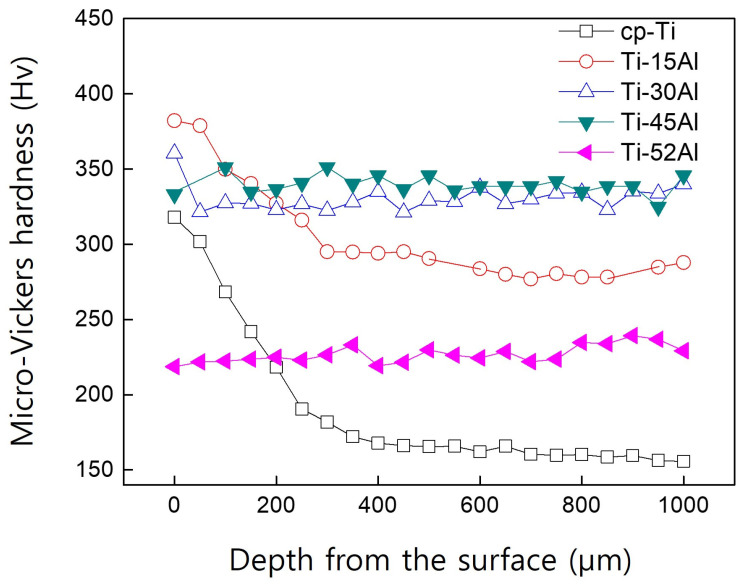
Micro-Vickers hardness depth profiles of Ti-xAl alloys. Open symbols indicate samples with an alpha-case layer, whereas closed symbols correspond to alloys without a distinct alpha-case. (Each data point represents the average of three measurements, and the standard deviations are smaller than the symbol size and therefore not shown).

Figure 4 presents the micro-Vickers hardness profiles obtained from the surface to the interior. Open symbols indicate samples exhibiting an alpha-case layer, while closed symbols correspond to alloys in which no distinct reaction zone was detected. The alpha-case thickness was defined as the depth where the hardness returned to the matrix level. For cp-Ti, the alpha-case reached approximately 350 μm, whereas it decreased sharply with increasing aluminum content and became negligible above 30 at% Al. Each hardness value represents the average of three measurements taken at different locations, and the standard deviations were smaller than the symbol size; therefore, error bars are not shown in the figure.

These findings reveal that the alpha-case thickness is inversely correlated with aluminum content. The suppression effect can be attributed to the reduced oxygen solubility and diffusivity in aluminum-rich alloys, which lower the driving force for oxide formation. Moreover, aluminum enrichment at the interface decreases the chemical activity of titanium, thereby minimizing the reaction between the melt and the mold materials.

### 3.3. Composition and Phase Analysis of the Alpha-Case

**Figure 5:**
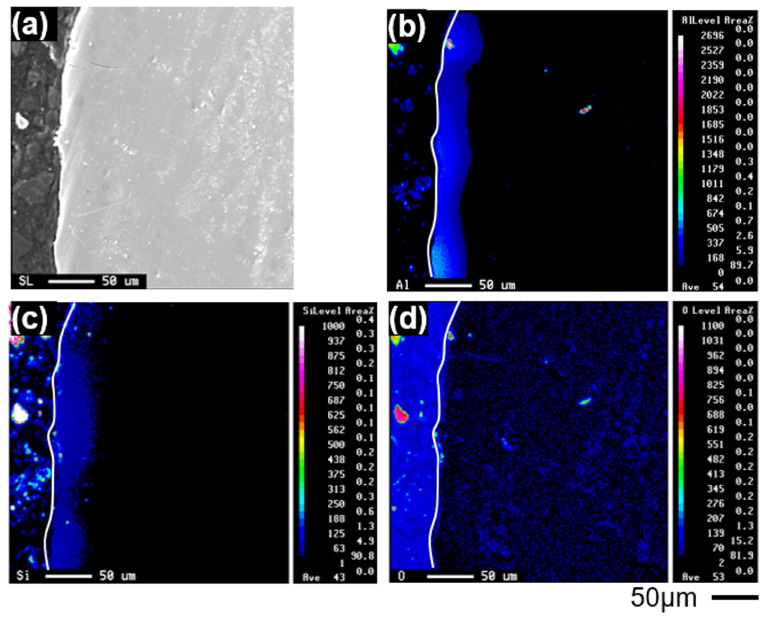
EPMA elemental maps near the surface of a cp-Ti casting: (**a**) optical micrograph of the mapped region, (**b**) aluminum, (**c**) silicon, and (**d**) oxygen distributions.

EPMA elemental maps of aluminum, silicon, and oxygen near the surface of cp-Ti castings are shown in Figure 5. Although a quantitative oxygen gradient could not be directly obtained due to instrumental limitations, the spatial distribution of aluminum and silicon was clearly identified up to a depth of approximately 50 μm.

**Figure 6:**
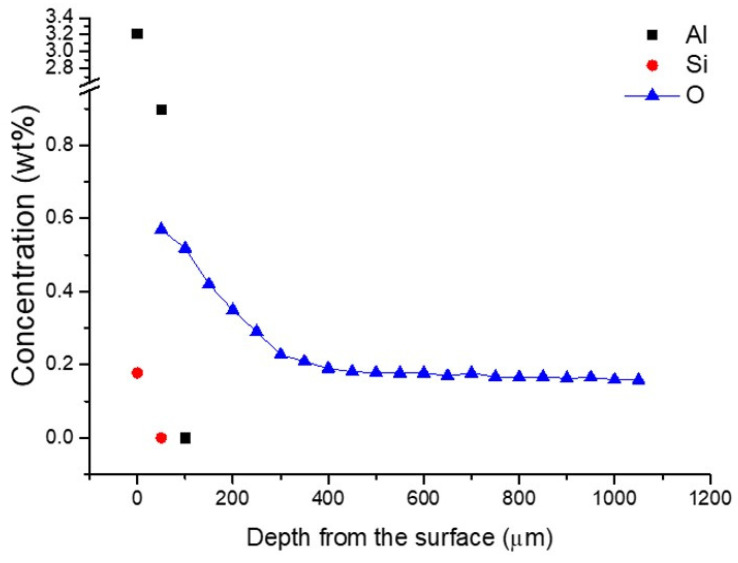
Depth profiles of aluminum and silicon concentrations measured by EDS, and oxygen concentration estimated from micro-Vickers hardness values. (The error of the chemical analysis is smaller than the size of the symbols).

To estimate oxygen concentration indirectly, micro-Vickers hardness values were converted based on the correlation proposed by Saha et al. [17,18], who demonstrated a strong linear relationship between oxygen content and hardness in titanium alloys. Figure 6 presents the depth profiles of aluminum and silicon concentrations measured by EDS, along with the oxygen concentration estimated from the hardness data. The nominal oxygen level in the cp-Ti used in this study was 0.20 wt%, while the measured oxygen concentration near the surface increased to about 0.21 wt% at a depth corresponding to the observed alpha-case boundary (~350 μm).

### 3.4. Thermodynamic Aspects of Alpha-Case Formation

The tendency for alpha-case formation decreased steadily with increasing aluminum content, and no distinct reaction layer was observed above 30 at% Al. This behavior reflects the reduction in the chemical activities of titanium and aluminum, both of which show pronounced negative deviations from Raoult’s law. Such deviations lower the chemical potentials of the constituent elements and contribute to the thermodynamic stabilization of Ti-Al alloys [19,20].

**Figure 7:**
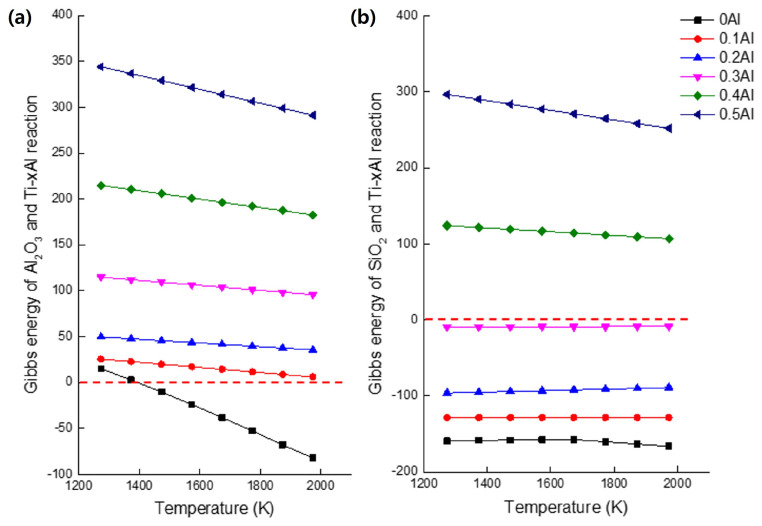
Thermodynamic analysis of Ti-xAl alloys showing (**a**) the Gibbs free-energy change for the reaction with Al_2_O_3_, and (**b**) that for the reaction with SiO_2_. The figure was redrawn and reprocessed by the authors based on the data and thermodynamic relationships reported in Refs. [19,20].

The thermodynamic basis for interpreting the interfacial reactions shown in Figure 7 can be expressed through the generalized reduction of mold oxides by the Ti-Al melt, as proposed by Kostov et al. [19]:

(1) $$M_{x}O_{y}\;\overset{\mathrm{liquid}\;\mathrm{Ti}-\mathrm{Al}}{\to }\;y\;O(\mathrm{Ti}\text{-}\mathrm{Al})\;+\;x\;M$$

This formulation describes oxygen transfer from the mold oxide to the Ti-Al melt and the concurrent reduction of the metallic element M. The Gibbs free-energy changes in Figure 7a,b were evaluated following the thermodynamic treatment of Kostov et al. [19,20], in which the Ti-Al melt is regarded as a non-ideal solution exhibiting negative deviations from Raoult’s law. The activities of titanium and aluminum were taken from established thermodynamic descriptions of the Ti-Al system in the literature. Under these assumptions, a positive Gibbs free energy of reaction signifies that the oxide remains stable against reduction, whereas a negative value indicates thermodynamically favorable reduction by the alloy.

Thermodynamic calculations showed that only the reaction between alumina and cp-Ti is spontaneous, whereas the reduction of silica becomes non-spontaneous for alloys containing more than 30 at% Al. The decreasing chemical activities of Ti and Al with increasing aluminum content stabilize the metal–mold interface and diminish the driving force for oxide formation. As a result, the interfacial reaction between Ti-xAl alloys and alumina molds weakens progressively with increasing Al, thereby suppressing alpha-case development.

Although the Gibbs free energy for alumina formation is lower than that for silica, the chemical enrichment in the alpha-case layer was dominated by aluminum. This trend arises from the high alumina content in the mold, which provides a far larger reservoir of aluminum at the interface. Titanium also shows a stronger thermodynamic affinity toward alumina than toward silica, further promoting aluminum-rich reaction products. Consequently, the compositional gradient across the reaction zone reflects both the abundance of alumina in the mold and the preferential interaction between titanium and alumina.

Overall, the suppression of alpha-case formation with increasing aluminum content is governed by the thermodynamic stabilization of the metal–mold interface. Higher aluminum levels reduce the chemical activity of titanium, lowering its affinity for oxygen and decreasing the driving force for interfacial oxidation. Thus, both mold composition and alloy chemistry exert decisive control over the thermodynamic stability and reactivity during investment casting of Ti-Al alloys.

### 3.5. Diffusion Aspects of Alpha-Case Formation

The reactivity between titanium alloys and oxide molds can also be interpreted from a diffusion perspective, considering oxygen as the dominant element controlling the growth of the alpha-case layer. The diffusion of oxygen in titanium alloys follows Fick’s second law, which can be expressed as:

(2) $$\frac{\partial C}{\partial t}=\frac{\partial }{\partial x}\left(D\frac{\partial C}{\partial x}\right)$$

where C is the oxygen concentration, *t* is time, *x* is distance from the surface, and *D* is the diffusion coefficient. Under the boundary conditions $C\left(0,t\right)=C_{0}$ and $C\left(\infty ,t\right)=C_{\infty }$, the oxygen concentration profile during solidification can be represented by:

(3) $$C=C_{0}-\left(C_{0}-C_{\infty }\right)\mathrm{erf}(\frac{t_{a}}{2\tau^{1/2}})$$

where $t_{a}$ is the alpha-case thickness and τ is the scaled time given by:

(4) $$\tau =\int_{0}^{t}D\;dt$$

The diffusion coefficient is temperature dependent and follows the Arrhenius relationship:

(5) $$D=D_{0}\exp\left(\frac{-Q}{RT}\right)$$

where $D_{0}$ is the pre-exponential factor, Q is the activation energy for diffusion, R is the gas constant, and T is the absolute temperature.

The thickness of the alpha-case corresponding to a critical oxygen concentration ($C^{*}$) can then be expressed as:

(6) $$t_{a}=2\lambda^{\prime }\tau^{1/2}$$

where $\lambda^{\prime }=erf^{-1}\left[\frac{C_{0}-C^{*}}{C_{0}-C_{\infty }}\right]$.

The relationship between the alpha-case thickness and cooling rate (*C_R_*) can be represented as:

(7) $$t_{a}=kC_{R}^{-1/2}$$

(8) $$k=2\mathrm{er}f^{-1}\left[\frac{C_{0}-C^{*}}{C_{0}-C_{\infty }}\right]I^{\frac{1}{2}}$$

(9) $$I=\int_{T_{0}}^{T_{L}}D_{0}\exp\left(\frac{-Q}{RT}\right)dT$$

(10) $$C_{R}=\frac{\Delta T}{\Delta t}=\frac{T_{L}-25\;{}^{\circ}C}{t}$$

**Figure 8:**
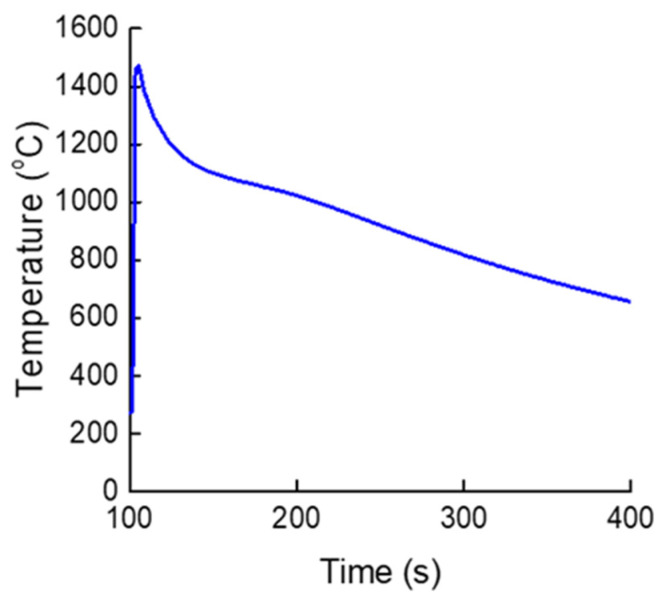
Representative temperature profile of Ti-xAl castings measured during solidification using a B-type thermocouple (Temperatures shown in °C).

According to these relationships, the alpha-case thickness is governed by temperature, activation energy, and cooling rate during solidification. Because all castings in this study were fabricated under identical conditions, the cooling rate effect was considered negligible, allowing the diffusion behavior to be treated as the dominant variable. The temperature variation of the Ti-xAl alloys during casting, measured using a thermocouple, is shown in Figure 8, which provides the thermal profiles used to calculate the diffusion parameters and reaction time between the molten metal and the mold. Because all Ti-xAl alloys exhibited essentially similar thermal behavior under the identical casting conditions employed in this study, the curve in Figure 8 is presented as a representative cooling profile. Although the alloys contained different Al contents, their cooling behaviors were effectively identical under these conditions and did not alter the overall trend relevant to diffusion-controlled alpha-case formation. Although oxygen uptake from the ceramic mold primarily occurs while the alloy is in the liquid state, the subsequent growth of the alpha-case layer is governed by solid-state oxygen diffusion during cooling. Accordingly, the thermal histories shown in Figure 8 provide the appropriate basis for the diffusion analysis described in this subsection.

To interpret the role of alloy phase structures, the diffusion coefficients ($D_{0}$) and activation energies (Q) for oxygen in different titanium phases were taken from literature and are summarized in Table 1. Oxygen diffusion is relatively fast in the β phase owing to its open body-centered cubic structure, whereas diffusion in the ordered α_2_ and γ phases is considerably slower due to their higher atomic packing and stronger Ti-Al bonding. This difference in diffusion kinetics explains the experimentally observed reduction in alpha-case thickness with increasing aluminum content.

**Figure 9:**
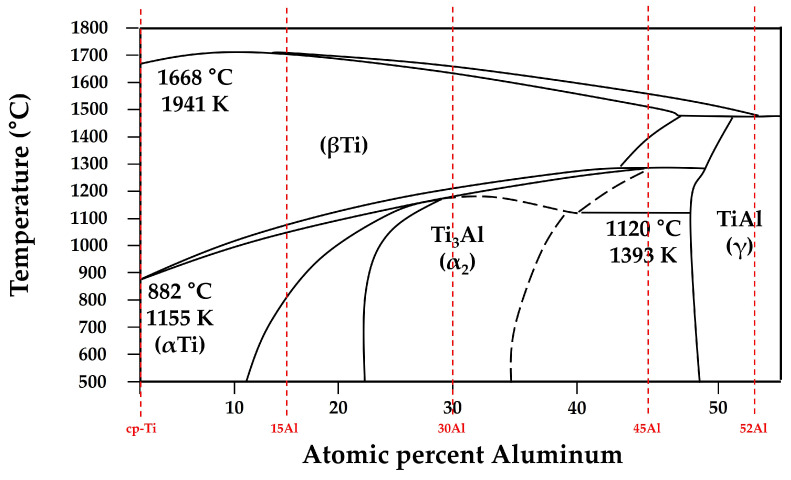
Ti-Al binary phase diagram redrawn and adapted from Okamoto et al. [24], with the alloy compositions examined in this study indicated by vertical dashed lines. The corresponding T_0_ and T_L_ values used in Table 2 were obtained from this diagram.

To establish the thermodynamic basis for the temperatures used in the diffusion calculations, the Ti-Al binary phase diagram is presented in Figure 9 [24] with the alloy compositions examined in this study indicated by vertical dashed lines. For each alloy, T_L_ was taken as the liquidus temperature corresponding to the onset of complete melting, whereas T_0_ was selected as the temperature at which solid-state phase stability (α, α_2_, or γ) begins, marking the transition from liquid-state oxygen uptake to diffusion-controlled oxygen transport into the solidifying matrix. These temperatures were extracted directly from the phase equilibria shown in Figure 9 and provide the thermodynamic framework for evaluating the integral term I in Equation (9) and the diffusion time τ. Based on these diffusion parameters, the calculated alpha-case thicknesses and corresponding reaction temperatures for the Ti-xAl alloys are summarized in Table 2. The calculated results show excellent agreement with experimental measurements, confirming that the reduction in alpha-case thickness is primarily attributed to decreases in oxygen diffusivity and effective reaction time at lower interfacial temperatures.

In summary, aluminum addition reduces the chemical activity of titanium (as demonstrated in Section 3.4.) and simultaneously stabilizes ordered intermetallic phases with low oxygen diffusivity. These combined thermodynamic and kinetic effects effectively suppress alpha-case formation, thereby improving the interfacial stability of titanium alloys during investment casting.

## 4. Conclusions

This study investigated the alpha-case formation behavior of Ti-xAl alloys during investment casting using oxide-based molds, considering both thermodynamic and diffusion aspects. The following conclusions were drawn.

The thickness of the alpha-case layer decreased markedly with increasing aluminum content. Alloys containing more than 30 at% aluminum exhibited negligible reactivity with the mold, and no distinct alpha-case layer was detected. This result demonstrates that aluminum addition effectively suppresses interfacial reactions during titanium alloy casting.

Elemental and phase analyses revealed that the alpha-case formed on cp-Ti consisted mainly of TiO_2_, Ti_3_Al, and Ti_5_Si_3_ phases. Among the constituent elements, oxygen exhibited the greatest diffusion depth, followed by aluminum, owing to its higher concentration in the alumina-based mold compared to silicon. These findings confirm that both the alumina mold and silica binder actively participated in the reaction with molten titanium.

From a thermodynamic viewpoint, increasing aluminum content reduced the chemical activities of both titanium and aluminum, thereby lowering the driving force for oxidation and improving interfacial stability. From a diffusion viewpoint, the reduction in oxygen diffusivity with increasing aluminum content was attributed to changes in diffusion coefficient, activation energy, and effective reaction temperature. The close agreement between calculated and measured alpha-case thicknesses supports this interpretation.

Overall, these results demonstrate that aluminum addition plays a dual role in suppressing alpha-case formation: it stabilizes the Ti-Al system thermodynamically and simultaneously restricts oxygen diffusion kinetically. The present findings provide valuable guidance for alloy design and process optimization to minimize interfacial reactions during the investment casting of titanium alloys.

## Figures and Tables

**Table 1:** Diffusion coefficients (D_0_) and activation energies (Q) for oxygen in various titanium phases [21,22,23].

Matrix	D_0_ (m^2^/s)	Q (kJ/mol)
β [21]	1.4 × 10^−5^	138
α [21]	8.9 × 10^−4^	220
γ [22]	1.6 × 10^−6^	128
α_2_ [23]	4.7 × 10^−9^	187

**Table 2:** Calculated alpha-case thicknesses and corresponding initial (T_0_) and reaction (T_L_) temperatures for Ti-xAl alloys in oxide-based molds.

Materials	Alpha-Case (μm)	T_0_ (K)	T_L_ (K)
cp-Ti	350	1155	1941
Ti-15Al	240	1333	1941
Ti-30Al	100	1500	1900
Ti-45Al	30	1393	1764
Ti-52Al	40	1155	1729

## Data Availability

The original contributions presented in the study are included in the article. Further inquiries can be directed to the corresponding author.
